# Journal editors: How do their editing incomes compare?

**DOI:** 10.12688/f1000research.25620.3

**Published:** 2021-02-01

**Authors:** Janice C. L. Lee, Jennifer Watt, Diane Kelsall, Sharon Straus

**Affiliations:** 1 University of Toronto, Toronto, Canada; 2 Unity Health Toronto, Toronto, Canada; 3 CMAJ Open and the CMAJ Group, Ottawa, Canada

**Keywords:** Journal, Medical Journalism, Peer review, Publishing, Income

## Abstract

**Background:** The work of journal editors is essential to producing high-quality literature, and editing can be a very rewarding career; however, the profession may not be immune to gender pay gaps found in many professions and industries, including academia and clinical medicine. Our study aimed to quantify remuneration for journal editors from core clinical journals, determine if a gender pay gap exists, and assess if there are remuneration differences across publishing models and journal characteristics.

**Methods:** We completed an online survey of journal editors with substantial editing roles including section editors and editors-in-chief, identified from the Abridged Index Medicus “Core Clinical” journals in MEDLINE. We analyzed information on demographics, editing income, and journal characteristics using a multivariable partial proportional odds model for ordinal logistic regression.

**Results:** There were 166 survey respondents (response rate of 9%), which represented editors from 69 of 111 journals (62%). A total of 140 fully completed surveys were analyzed (95 males and 45 females); 50 (36%) editors did not receive remuneration for editorial work. No gender pay gap and no difference in remuneration between editors who worked in subscription-based publishing vs. open access journals were detected. Editors who were not primarily health care providers were more likely to have higher editing incomes (adjusted odds ratio [OR] 2.96, 95% confidence interval [CI] 1.18-7.46). Editors who worked more than 10 hours per week editing earned more than those who worked 10 hours or less per week (adjusted OR 16.7, 95%CI 7.02-39.76).

**Conclusions:** We were unable to detect a gender pay gap and a difference in remuneration between editors who worked in subscription-based publishing and those in open access journals. More than one third of editors surveyed from core clinical journals did not get remunerated for their editing work.

## Introduction

The number of academic journals continues to grow each year. In 2018, there were 5399 clinical journals tracked by Journal Citation Reports in comparison to only 3681 10 years prior ^1^. A rise in open-access journals is also evident; while in 2008 there were just 249 open-access journal titles in Web of Science, that number ballooned to 1431 in 2018 ^1^. Growth in the journal industry comes with more opportunities to become a journal editor. Editors are often scientists, researchers, administrators, and clinicians who have expertise in a particular field and competencies in evaluating articles’ suitability for publication in scientific journals ^2^. Editorial work can be a very satisfying full-time career or part-time job in addition to a clinical, research, or administrative careers.

Within medicine, substantial evidence of gender inequity in academia exists, including disparities in compensation ^3– 5^. For example, a 2012 US survey found that male physician researchers had higher average salaries ($13,399 USD; p=0.001) than females after adjusting for specialty, academic rank, and research productivity ^6^. Other studies have shown salary deficits can be up to 12–28% for female physicians ^7, 8^ when compared to male counterparts. Similar statistics can be found in the UK, where the average pay gap for consultant physicians, including professors, was 13% in favor of men ^9^. Several countries have mandated pay transparency laws in recent years. Denmark ^10^ and Austria ^11^ passed laws requiring companies to internally report gender-based wage statistics to it employees in 2006 and 2010 respectively. In 2018, the United Kingdom pushed for national pay transparency and equity by mandating public annual reporting of gender pay gap for any organization comprising more than 250 employees ^12^. France followed suit with mandatory reporting, while Germany and Iceland enacted pay equity and transparency laws.

While there is an expanding body of literature on pay gaps in of academia and of medicine in general ^9, 13^, there is currently very limited literature that evaluates how journal editors are compensated for their work. An international email survey of 88 editors of nursing journals found that their mean annual salary was $12,749 USD (ranging from $0 to $56,000) for a mean of 13.4 hours worked per week ^14^. A total of 8% of survey respondents (7 of the 88 editors) did not receive any monetary compensation and only 31% of participants felt that their compensation was adequate ^14^. It is concerning that the critical job of editors to uphold the integrity of academic literature can be low-paid or voluntary. With mandatory data reporting, The Lancet reported gender pay gaps of 13–40% favouring men over women in major UK publishing companies in 2018 ^15^. Specific data for journal editors were not available but some of these data do highlight the potential gaps.

There is substantial heterogeneity among journals, such as publishing platform, scope, publication frequency, and Journal Impact Factor ^16^. Though open access publishing comes at a large cost to authors, its popularity is supported by many benefits that may include faster publishing times and the ability to reach bigger audiences compared to subscription-based publishing. It is unknown whether a journal editor’s remuneration is affected by these journal variables.

With special interest in biomedical sciences, the objectives of this study were to quantify remuneration for journal editors from core clinical journals, determine if a gender pay gap exists, and assess if there are remuneration differences across publishing models (e.g., subscription-based or open-access) and other journal characteristics (e.g., publication frequency and Journal Impact Factor).

## Methods

### Design and participant recruitment

We completed an international online survey of full-time and part-time journal editors identified from the Abridged Index Medicus “Core Clinical” journals in MEDLINE, which represented 111 peer-reviewed core medical journals with high clinical impact. Our target population of journal editors were those with substantial editing roles including editors-in-chief, deputy editors, executive editors, senior editors, associate editors, and editors of a specialty section. Editors were identified through each journal’s webpage in December 2018. We excluded roles classified as statistical editors, assistant editors, international editors and editorial board members. If the editors of a section or specialty section numbered greater than 200 for a single journal, these individuals were excluded based on the assumption that they may not have a substantial editorial role. Publicly available emails were found via the English-language search engine, Google. Major sources included journal web pages, academic institutional web pages, and corresponding author on recently published articles. Reporting of this online survey was guided by the CHERRIES reporting guideline ^17, 18^.

### Survey development and pre-testing

The online survey was conducted using the subscription software, Qualtrics CoreXM Survey Tool ^19^. We developed the survey to capture demographic data, editing remuneration in USD, editing experience, and journal characteristics. A blank copy of the survey is available as *Extended data* ^18^. An online pilot test was sent out to our knowledge user team of three journal editors to identify poorly constructed questions, and to assess face validity before distribution. The survey included 10 questions on three pages, and adaptive questioning was used. Respondents were able to review their answers before submission. The following variables were collected in our survey and included in the final multivariable model: sex (male vs. female), gender identity, primary role (health care provider vs. other), academic rank (any professorship vs. none), editing role (section/specialty/associate editor vs. editor-in-chief/executive/senior/deputy editor/other), years in editing (>10 vs. ≤10 years), hours/week in editing role (>10 vs. ≤10 hours), and years worked for current journal (>5 vs. ≤5 years). Journal characteristics of publishing model (subscription-based, vs. open or hybrid [open access option or open access for developing countries]) and frequency of publication (best fitted to monthly/bimonthly vs. weekly/biweekly) were extracted from the journal selected in the survey. A journal’s 2017 Journal Impact Factor was obtained from Journal Citation Reports ^1^.

### Survey administration

Email invitations with the survey link were distributed via Qualtrics in February 2019. This was a voluntary survey, and no incentives were offered. We employed established methods to enhance survey completion rates with reminder emails at week 2 and week 4 ^20^.

### Statistical analysis

Only completed surveys were analyzed. Dichotomous baseline characteristics for male and female journal editors were presented as frequencies and percentages and compared with the chi-square test or Fisher’s exact test when expected sample sizes were 5 or less. Journal Impact Factor of the journal where male and female editors worked was a non-normally distributed continuous variable presented as a median with interquartile range. The Journal Impact Factor of each group was compared with the Wilcoxon rank sum test.

Our outcome of interest was journal editor salary, which was modeled as an ordinal variable with three categories: ≤$10,000 per year, $10,001 to $50,000 per year, and ≥$50,000 per year. We derived adjusted odds ratios (OR) and 95% confidence intervals (CI) for our outcome of interest from a multivariable partial proportional odds model for ordinal logistic regression in SAS version 9.4 (SAS Institute, Cary, North Carolina). The variables for editor sex and academic appointment did not satisfy the proportional odds assumption; therefore, these variables were assumed to have nonproportional odds in the final multivariable partial proportional odds model. Two-sided p-values were reported and p-values <0.05 were considered statistically significant.

### Ethical approval

Institutional review board approval was obtained through the Unity Health Toronto Research Ethics Board in Toronto, Canada. The survey landing page included study information and consent to participate was implied by survey completion and submission.

## Results

**Figure 1.**
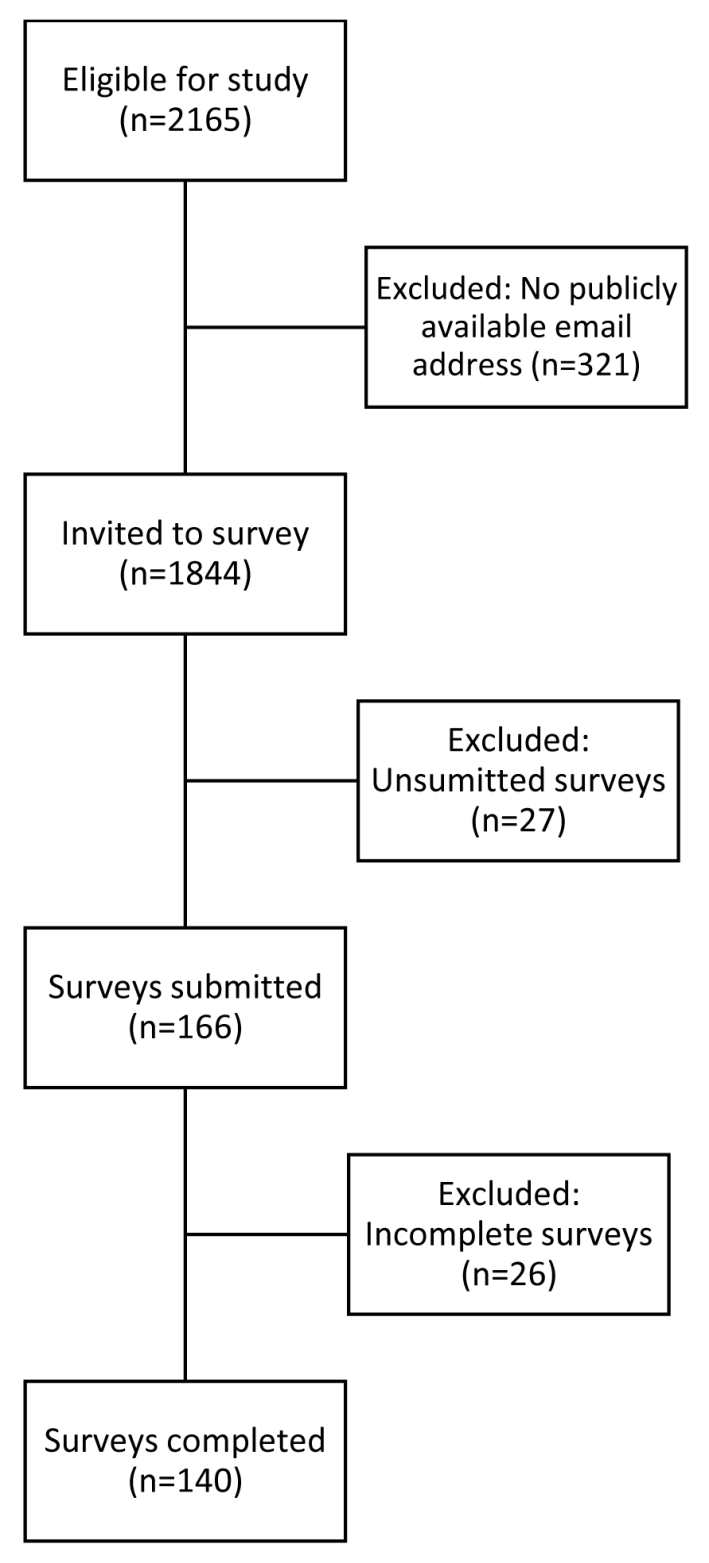
Diagram of study recruitment.

A total of 2165 editors were identified in December 2018, with 1948 having publicly available email addresses; of these, survey invitations were successfully sent to 1844 without bounce-back. A total of 193 surveys were started, and 166 surveys were submitted (overall response rate of 9%). We received survey responses from journal editors at 69 of 111 journals (62%). 140 completed surveys were included in our analyses ( Figure 1). Data that had small cell counts (n<5) were not reported to preserve the privacy of participants. A de-identified version of the dataset is available as Underlying data ^18^. This was composed of 95 male and 45 female editors. All respondents identified as cis gender. A total of 50 (36%) editors did not receive remuneration for editorial work; 111 (79%) and 29 (21%) editors worked for a subscription-based journal versus an open/hybrid journal, respectively. A total of 90% of editors held an academic position. The median 2017 Journal Impact Factor was 4.9 (Interquartile range [IQR] 3.5-6.6 for males and 3.5-7.5 for females). There was a larger proportion of female survey respondents who were fulltime journal editors compared to male counterparts (10 females [22%] vs. 4 males [4%] p=<0.01). Details on baseline characteristics are provided in Table 1.

**Table 1.** Baseline characteristics of survey respondents, by sex.

Characteristic	Females (n=45)	% of Females	Males (n=95)	% of Males	p-value ^*^
**Annual remuneration in USD, n**					0.01
$10000 or less	24	53.3%	67	70.5%	
$10001–50000	7	15.6%	20	21.1%	
Greater than $50000	14	31.1%	8	8.4%	
**Publishing Model, n**					0.1
Subscription-based	32	71.1%	79	83.2%	
Hybrid or Open Access	13	28.9%	16	16.8%	
**Primary Role/Occupation, n**					<0.01
Health Care Provider	11	24.4%	48	50.5%	
Other	34	75.6%	47	49.5%	
**Academic Appointment, n**					0.07 ^**^
None	8	17.8%	6	6.3%	
Assistant, Associate, Full Professor or Professor Emeritus	37	82.2%	89	93.7%	
**2017 Journal Impact Factor,** **median (IQR)**					0.53 ^***^
	4.9 (3.5-7.5)		4.9 (3.5-6.6)		
**Frequency of Publication, n**					0.21
Monthly or Bimonthly	29	64.4%	71	74.7%	
Weekly or Biweekly	16	35.6%	24	25.3%	
**Editing Role, n**					0.26
Section/Specialty/Associate Editor	30	66.7%	72	75.8%	
Editor-in-Chief/Executive/ Senior/ Deputy Editor/Other	15	33.3%	23	24.2%	
**Cumulative Years of Experience** **in Editing, n**					0.08
10 or less	29	64.4%	46	48.4%	
Greater than 10	16	35.6%	49	51.6%	
**Hours Spent Per Week Editing, n**					<0.01
10 or less	27	60.0%	79	83.2%	
Greater than 10	18	40.0%	16	16.8%	
**Years Employed at the Journal, n**					0.52
5 or less	23	51.1%	43	45.3%	
Greater than 5	22	48.9%	52	54.7%	

USD = United States dollar. IQR = Interquartile range. * chi-square test. **Fisher’s exact test. ***Mann-Whitney U test.

In univariate analyses ( Table 2), journal editors received more remuneration if the journal was open/hybrid access rather than subscription-based (OR 3.06, 95% CI 1.39-6.75), if the editor was not primarily a health care provider (OR 3.67, 95% CI 1.7-7.91), if the issues were weekly/biweekly rather than monthly/bimonthly (OR 2.07, 95% CI 1-4.28), if the editors held senior editing positions (OR 4.5, 95% CI 2.16-9.59), if the editors spent more than 10 hours per week editing (OR 16.7, 95% CI 7.02-39.76), and if the editors had worked for more than 5 years at the journal (OR 2.28, 95% CI 1.13-4.62).

**Table 2.** Association between editor characteristics and remuneration.

Characteristics	Unadjusted OR (95% CI)	Adjusted OR ^*^(95% CI)
Female sex		1.39 (0.56 to 3.41)
Annual remuneration of greater than $50000 USD: Females vs. males	4.91 (1.88 to 12.83)	
Annual remuneration $10,000 to $50,000 USD: Females vs. males	2.09 (1.01 to 4.36)	
Publishing model: Hybrid or Open Access publishing, vs. Subscription-based	3.06 (1.39 to 6.75)	0.83 (0.26 to 2.66)
Primary occupation: non-health care provider, vs. health care provider	3.67 (1.7 to 7.91)	2.96 (1.18 to 7.46)
Academic appointment vs. none		0.43 (0.1 to 1.92)
Annual remuneration of greater than $50,000 USD: Any professorship, vs. none	0.09 (0.03 to 0.31)	
Annual remuneration of $10,500 to $50,000 USD: Any professorship, vs. none	0.26 (0.08 to 0.82)	
2017 Journal Impact Factor, median (IQR)	1.06 (1.02 to 1.11)	1.02 (0.96 to 1.09)
Frequency of Publication: weekly/biweekly, vs. monthly/bimonthly	2.07 (1 to 4.28)	1.45 (0.51 to 4.16)
Editing Role: Editor-in-Chief/Executive/Senior/Deputy/Other, vs. Section/ Specialty/Associate editors	4.5 (2.16 to 9.59)	1.88 (0.66 to 5.32)
Cumulative Years of Experience in Editing: greater than 10, vs. 10 or less	1.96 (0.98 to 3.9)	1.87 (0.74 to 4.77)
Hours Spent Per Week Editing: greater than 10, vs. 10 or less	16.7 (7.02 to 39.76)	10.33 (3.32 to 32.11)
Years Employed at the Journal: greater than 5, vs. 5 or less	2.28 (1.13 to 4.62)	2.22 (0.85 to 5.77)

USD = United States dollar. IQR = Interquartile range. CI = Confidence interval. OR = odds ratio. *Adjusted OR for all characteristics in the table.

In multivariable analysis ( Table 2), there was no gender pay gap detected or remuneration differences identified between publishing models. We found that editors who were not primarily health care providers were more likely to have higher editing incomes (adjusted OR 2.96, 95%CI 1.18-7.46). Editors who worked more than 10 hours per week editing earned more than those who worked 10 hours or less per week (adjusted OR 16.7, 95%CI 7.02-39.76).

## Discussion

In addition to a recent study by *The Lancet* highlighting the presence of a gender pay gap of employees at major academic publishing companies ^15^, numerous studies in the past have highlighted similar inequities across academia ^3– 8^. In our study of part-time and full-time journal editors from a focused subset of core clinical journals, we did not detect a gender pay gap. We found that annual remuneration for editing was higher for editors working more than 10 hours per week and if their primary occupation was not a health care provider. Full-time journal editing positions were disproportionately more likely to be held by a woman. While we hypothesized that the different revenue structure of subscription-based versus open access journals may translate to a difference in editor’s remuneration, we did not find it to be true after adjusting for predictor variables. A journal’s Impact Factor did not affect an editor’s remuneration. Although 36% of editors surveyed reported no direct earnings from their editorial work, there can be other benefits including subsidies for scientific meeting registration fees and travel costs.

### Limitations

While there is robust data on gender pay gaps in academia ^13^, our study only examined gaps specifically in biomedical sciences. Our study had several limitations. First, our results had limited generalizability due to a low response rate, which can magnify volunteer bias. However, our participants did represent 69% of the core clinical journals. Moreover, our sample size of 140 was comparable to an international survey of 148 scientific editors from biomedical journals to evaluate journal editing core competencies ^21^. We were unfortunately not powered to compare subgroups of editing roles and journal characteristics. Second, given our approach to identifying email addresses for journal editors, our survey was biased towards editors who had either an English-language academic profile on an institutional website or if they were a corresponding author of a recently published article. Third, we were unable to make direct comparisons between for-profit and non-profit journals, because several companies publish journals on behalf of non-profit organizations. This distinction may factor into a journal editor’s desire to edit for a journal at a given remuneration rate. In addition, we did not assess non-financial reasons that factor into the decision to be a journal editor. Lastly, there was substantial heterogeneity in editing roles and the lack of standardization in editing titles made it difficult to draw conclusions that were generalizable to the population. Future studies can include qualitative interviews to gauge the various roles and responsibilities of editors, and remuneration practices.

## Conclusion

We conducted an international survey of journal editors from core clinical journals to understand how remuneration varied across editor’s demographics, professional experience, and journal characteristics. We did not detect a gender pay gap or a difference in remuneration between editors who worked in subscription-based publishing vs. open access journals. More than a third of editors surveyed were not remunerated for their work.

## Data availability

### Underlying data

Harvard Dataverse: Journal editors: How do their editing incomes compare? https://doi.org/10.7910/DVN/AHMB8G ^18^.

File ‘Journal editors deidentified data.tab’ contains a de-identified version of the dataset generated in this study.

Due to the nature of this research, data provided is a limited de-identified dataset without potentially identifying information, i.e. clinical specialty (if applicable) and journal characteristics.

Individual(s) wishing for access to the full de-identified dataset requires written support from the principal investigator (Dr. Sharon Straus, the corresponding author) to be added to the study as study personnel(s), and subsequently needs approval from the Unity Health Toronto Research Ethics Board in Toronto, Canada.

### Extended data

Harvard Dataverse: Journal editors: How do their editing incomes compare? https://doi.org/10.7910/DVN/AHMB8G ^18^.

File ‘Journal Editors Supplementary 2 - Survey.docx’ contains a copy of the survey used in this study.

### Reporting guidelines

Harvard Dataverse: CHERRIES checklist for ‘Journal editors: How do their editing incomes compare?’. https://doi.org/10.7910/DVN/AHMB8G/XPMGJL ^18^.

Data are available under the terms of the Creative Commons Zero “No rights reserved” data waiver (CC0 1.0 Public domain dedication).
