# Supplementary material for: Journal editors: How do their editing incomes compare?
Source: F1000Res. 2021 Feb 1;9:1027. Originally published 2020 Aug 24. [Version 3] doi: 10.12688/f1000research.25620.3 (PMC7836083; doi:10.12688/f1000research.25620.3)
Supplement: Peer review history

# Peer review: Reviewer response for version 3

**DOI:** 10.5256/f1000research.54238.r78579

**Published:** 2021-02-01

**Authors:** Carl Singleton

**Affiliations:** 1 Department of Economics, University of Reading, Reading, UK

Amendments are minor and do not change my previous view of "Approving" the article.

Is the work clearly and accurately presented and does it cite the current literature?

Yes

If applicable, is the statistical analysis and its interpretation appropriate?

Yes

Are all the source data underlying the results available to ensure full reproducibility?

Yes

Is the study design appropriate and is the work technically sound?

Partly

Are the conclusions drawn adequately supported by the results?

Partly

Are sufficient details of methods and analysis provided to allow replication by others?

Yes

Reviewer Expertise:

Labour Economics; Applied Econometrics, including published works on gender in the workplace and the gender pay gap.

I confirm that I have read this submission and believe that I have an appropriate level of expertise to confirm that it is of an acceptable scientific standard.

# Peer review: Reviewer response for version 2

**DOI:** 10.5256/f1000research.53885.r77562

**Published:** 2021-01-25

**Authors:** Kelly Cobey

**Affiliations:** 1 Investigator, Centre for Journalology, Ottawa Hospital Research Institute, Ottawa, ON, Canada

The authors have addressed the vast majority of my previous comments in their current version. 

 Minor point: The authors state "2017 Journal Impact Factor, and frequency of publication (best fitted to monthly/bimonthly vs. weekly/biweekly), were extracted from the journal selected in the survey." 

 I would specify where journal impact factor was extracted from - many journals no longer list this information on their website. In these cases was this coded as missing? Or, was the data searched for elsewhere?

Is the work clearly and accurately presented and does it cite the current literature?

Yes

If applicable, is the statistical analysis and its interpretation appropriate?

Yes

Are all the source data underlying the results available to ensure full reproducibility?

Yes

Is the study design appropriate and is the work technically sound?

Yes

Are the conclusions drawn adequately supported by the results?

Yes

Are sufficient details of methods and analysis provided to allow replication by others?

Partly

Reviewer Expertise:

Journalology; publication science; open science

I confirm that I have read this submission and believe that I have an appropriate level of expertise to confirm that it is of an acceptable scientific standard.

# Peer review: Reviewer response for version 2

**DOI:** 10.5256/f1000research.53885.r77564

**Published:** 2021-01-19

**Authors:** Carl Singleton

**Affiliations:** 1 Department of Economics, University of Reading, Reading, UK

I am satisfied that the authors have taken on board my comments, as far as possible given their current limited access to the data. The authors are also fair and transparent with their methods, assumptions and the potential limitations of the study.

Is the work clearly and accurately presented and does it cite the current literature?

Yes

If applicable, is the statistical analysis and its interpretation appropriate?

Yes

Are all the source data underlying the results available to ensure full reproducibility?

Yes

Is the study design appropriate and is the work technically sound?

Partly

Are the conclusions drawn adequately supported by the results?

Partly

Are sufficient details of methods and analysis provided to allow replication by others?

Yes

Reviewer Expertise:

Labour Economics; Applied Econometrics, including published works on gender in the workplace and the gender pay gap.

I confirm that I have read this submission and believe that I have an appropriate level of expertise to confirm that it is of an acceptable scientific standard.

# Peer review: Reviewer response for version 1

**DOI:** 10.5256/f1000research.28274.r75952

**Published:** 2020-12-11

**Authors:** Carl Singleton

**Affiliations:** 1 Department of Economics, University of Reading, Reading, UK

This study aimed to quantify gender differences in pay for editing services within academia, specifically for “Core Clinical” journals. It also aimed to test for the presence of a gender pay gap, conditional on other observable (surveyed) characteristics of editors and their journals.

 Overall, the article is well-written, the methods are clearly explained, and some of the limitations are fairly outlined.

 Cobey (2020) has already provided a thoughtful review of this article, and I would echo those comments.

 In addition, I would like to make the following further suggestions:

1. In the introduction and the limitations, the authors may wish to dwell on the other reasons that editors choose to do what they do, besides direct financial gain (e.g., enjoyment, prestige, compensation from their main employer in the form of relief from teaching or other activities). These motivations are all potentially endogenous factors (omitted variables) in the regression model. While in theory they may not be correlated with gender in the population of editors, the sample size here is small and the response to the survey probably non-random.
2. The literature cited for the gender pay gap is in academia is limited and very specific, focused on medical sciences. But there are good examples and literature reviews of gender pay gaps in academia more widely. One recent example I am aware of is Mumford & Sechel (2020) ^1^ in the *British Journal of Industrial Relations*. While that study focuses on the gender pay gap among economists, it also provides some prominent examples of the wider literature on the gender gaps in academia, including in Medicine and Science (e.g., Connolly and Holdcroft, 2009 ^2^). The authors could look to reference more of this previous work, particularly since this journal is not field-specific.
3. The authors state that “Only completed surveys were analyzed”. Elsewhere in the article it is mentioned that responses where editors “preferred not to say” were discarded. Having looked at the survey (Lee *et al.*, 2020), there are some questions asked that are not being used in the analysis. Therefore, the article could be clearer on what information is being thrown away. For instance, for the unadjusted odds ratios, I am not sure why an editor preferring not to say what the frequency of their publication is should exclude them from the sample when estimating the raw gender pay gap. Overall, it is important that the authors explain why they don’t consider a larger sample for their statistics just because of some non-response to some survey questions.
4. Like the previous comment, I am also concerned about the amount of information lost in the chosen modelling strategy. The survey collects information on much narrower bands of pay (and hours). While I appreciate that in this field odds models are common and often preferred, in my own field, as an applied economist/econometrician, we would prefer not to lose all the information contained in the pay data collected. For example, I would suggest also estimating a censored linear least squares regression model (i.e., tobit, censored because of the zeroes), where the mid-point of each and every range in the survey is imputed as the pay value, which becomes the dependent variable (normally in natural logs). Similarly, combining the narrow pay bands and narrower hours bands in the survey could allow the authors to look at a measure of hourly pay, which is what matters most economically, being the compensation for a unit of labour input (see Mumford and Smith, 2007 ^3^, for a relevant example of extracting more information from ordinal pay and hours data in this suggested way). Results following this alternative empirical strategy could be presented as a complement to those already shown in the article.
5. In the current ordinal logistic regression model approach, I would be curious what interacting gender with some the other covariates shows. For example, the estimation sample shows a large difference between the male and female in the proportion of editors whose main occupation is Health Care Provider. This variable looks as though it could be worth interacting with gender. This could be added as a third column of results in Table 2.

Minor comment:

1. The Introduction claims that the UK led the wag on gender pay gap reporting legislation. Denmark and Austria have legislation requiring firms to make their pay gaps available to their workers, which I believe substantially pre-date the UK policy (2006 in Denmark; see Bennedsen *et al*., 2019 ^4^).

Is the work clearly and accurately presented and does it cite the current literature?

Yes

If applicable, is the statistical analysis and its interpretation appropriate?

Yes

Are all the source data underlying the results available to ensure full reproducibility?

Yes

Is the study design appropriate and is the work technically sound?

Partly

Are the conclusions drawn adequately supported by the results?

Partly

Are sufficient details of methods and analysis provided to allow replication by others?

Yes

Reviewer Expertise:

Labour Economics; Applied Econometrics, including published works on gender in the workplace and the gender pay gap.

I confirm that I have read this submission and believe that I have an appropriate level of expertise to confirm that it is of an acceptable scientific standard, however I have significant reservations, as outlined above.

## References

[1] : Pay and Job Rank among Academic Economists in the UK: Is Gender Relevant?. *British Journal of Industrial Relations*.2020;58(1) : 10.1111/bjir.12468 82-113 10.1111/bjir.12468

[2] : The pay gap for women in medicine and academic medicine.2009;

[3] : THE GENDER EARNINGS GAP IN BRITAIN: INCLUDING THE WORKPLACE. *The Manchester School*.2007;75(6) : 10.1111/j.1467-9957.2007.01040.x 653-672 10.1111/j.1467-9957.2007.01040.x

[4] : Do Firms Respond to Gender Pay Gap Transparency?.2019; 10.3386/w25435 10.3386/w25435

# Response

**Published:** 2021-01-10

**Authors:** Janice CL Lee

**Affiliations:** University of Toronto, Canada

Re: #1) Thank you. Revised to address this limitation in both the introduction and limitations sections.

 Re: #2) Thank you. This was added to the limitations section. Mumford & Sechels 2020 and Connolly and Hodcroft 2009 were cited.

 Re: #3) Thank you. This was clarified in the manuscript. Of the 166 surveys, 140 were analyzed, excluding surveys that had incomplete responses. Responses of “prefer not to answer” were analyzed but often had small cell counts (n<5), and therefore the data were not reported to preserve privacy of participants.

 Re: #4) We did not perform the tobit regression, since we did not have exact salaries and cannot make an assumption regarding the mid-point values for the last category of salaries greater than $150,000 USD. In analyzing the zero/nonzero pay by male/female to explore censoring from the zeros, we found the following:

 chi-squared 1.35 (p=0.25)

 2x2 table:

 Zero salary, male: 37

 Zero salary, female: 13

 Nonzero salary, male: 58

 Nonzero salary, female: 32

 This analysis has not been included in the article.

 Re: #5) We presented the data based on male/female as primary exposure and did not examine for possible interaction of gender with covariates. We are unable to return to the data at this time to update the analysis to explore this possibility.

 Re: Minor #1) Thank you. This has been revised to add Denmark and Austria’s reporting legislation.

# Peer review: Reviewer response for version 1

**DOI:** 10.5256/f1000research.28274.r72508

**Published:** 2020-10-27

**Authors:** Kelly Cobey

**Affiliations:** 1 Investigator, Centre for Journalology, Ottawa Hospital Research Institute, Ottawa, ON, Canada

This study aimed to:

1. quantify remuneration for journal editors from core clinical journals
2. determine if a gender pay gap exists
3. assess if there are remuneration differences across publishing models and journal characteristics.

The authors addressed these aims through conducting an online survey of journal editors identified from the Abridged Index Medicus “Core Clinical” journals in MEDLINE.

 The paper is reported clearly and acknowledges relevant limitations. I describe a few suggestions for changes below:

- The authors specify:

*“The following variables were collected in our survey and included in the final multivariable model: sex (male vs. female), gender identity, publishing model (subscription-based, vs. open or hybrid [open access option or open access for developing countries]), primary role (health care provider vs. other), academic rank (any professorship vs. none), 2017 Journal Impact Factor, frequency of publication (monthly/ bimonthly vs. weekly/biweekly), editing role (section/specialty/ associate editor vs. editor-in-chief/executive/senior/deputy editor/other), years in editing (>10 vs. ≤10 years), hours/week in editing role (>10 vs. ≤10 hours), and years worked for current journal (>5 vs. ≤5 years) .”*

 When I review the survey provided by the authors, I don’t see any items asking about the 2017 journal impact factor or journal frequency of publication. Perhaps I missed something? If not, this leads me to think that perhaps the authors extracted this information online based on participants responses indicating which journal they are an editor at, or that the incorrect survey has been uploaded. This should be clarified in the paper.

- Further, the authors indicate the survey captured the frequency at which the included editors’ journals publish using the options: monthly/ bimonthly vs. weekly/biweekly. This is unlikely to apply uniformly across journals. For illustration, I don’t know how the F1000 journal editor would accurately respond to this item.
- Did the authors record bounce-backs among editors who they e-mailed their invitation to? In comparable work I have conducted this has been a fairly significant issue, especially when editor information is found via Google rather than through the journal website. If so, what was the N and could this be reflected in the study recruitment description and figure? The response rate may be somewhat higher if this is considered.

 
**Minor comments:**

- Given that this is not a sub-specialty journal, the authors could consider briefly addressing what the role and responsibilities of journal editors are in the introduction.
- The authors note: “The online survey was conducted using Qualtrics CoreXM Survey Tool software14, but an open access alternative such as SurveyMonkey15 could be used for replication of methods. ; I don’t think the free SurveyMonkey would be appropriate for replicating the survey described, I understand it allows for only 10 questions (each sub question ‘counts’) and 100 responses.
- The authors could provide rational for the journal sampling approach.

Is the work clearly and accurately presented and does it cite the current literature?

Yes

If applicable, is the statistical analysis and its interpretation appropriate?

Yes

Are all the source data underlying the results available to ensure full reproducibility?

Yes

Is the study design appropriate and is the work technically sound?

Yes

Are the conclusions drawn adequately supported by the results?

Yes

Are sufficient details of methods and analysis provided to allow replication by others?

Partly

Reviewer Expertise:

Journalology; publication science; open science

I confirm that I have read this submission and believe that I have an appropriate level of expertise to confirm that it is of an acceptable scientific standard, however I have significant reservations, as outlined above.

# Response

**Published:** 2021-01-10

**Authors:** Janice CL Lee

**Affiliations:** University of Toronto, Canada

Re: Extracting journal information--Yes, revised to state that journal information was extracted online based on journal selection in the survey.

 Re: Journal frequency—Journal frequency was extracted from journals and rounded up/down to fit the categories.

 Re: Bounce-back emails—Yes, revised to clarify that there were 104 bounce-backs. The response rate was calculated based on the number of successful emails sent.

 Re: Minor comment on role of journal editors—Thank you, this has been added to the introduction.

 Re: Minor comment on free survey software—Thank you, this has been removed. To our knowledge, there are no easily accessible free survey software for survey replication.

 Re: Minor comment on the rational for journal sampling—Revised to state that we chose the core clinical journal for their high clinical impact on medicine.
